# Masked ECG Changes in Wolff-Parkinson-White Syndrome Coexisting With Myocardial Infarction: A Case Report

**DOI:** 10.7759/cureus.64507

**Published:** 2024-07-14

**Authors:** Mustafa Çifci

**Affiliations:** 1 Emergency Medicine, University of Health Sciences, Kocaeli Derince Training and Research Hospital, Kocaeli, TUR

**Keywords:** myocardial infarction with no obstructive coronary atherosclerosis, high-sensitivity troponin t, echocardiography diagnosis, non-st elevated myocardial infarction, wolff-parkinson-white (wpw)

## Abstract

Wolff-Parkinson-White (WPW) syndrome, known for episodes of tachycardia and distinctive electrocardiographic (ECG) patterns, often makes it harder to diagnose myocardial infarction (MI) because it can hide the usual ECG signs of MI. Early use of high-sensitivity troponin levels and echocardiography to detect myocardial injury in WPW is important, facilitates timely intervention and improves patient outcomes. This report presents the case of a 39-year-old Caucasian male with no chronic disease history who presented to a family health center with intermittent mild chest pain localized to the left side, characterized by a burning and dull ache, for one week. On the day of presentation, the patient experienced increased pain accompanied by palpitations and mild sweating. An ECG at the family health center showed findings of WPW. Due to the presence of typical chest pain and WPW pattern on the ECG, the patient was referred to a tertiary hospital emergency department. At the tertiary hospital, repeat ECGs showed no changes, but blood tests revealed elevated troponin T levels (495 ng/ml initially, 485 ng/ml after 4 hours). The patient was admitted to the cardiology critical care ward. Echocardiography indicated regional wall motion abnormalities in specific segments. Coronary angiography revealed ectasia in vessels with slow flow but no obstructed vessels. This case underscores the diagnostic challenges posed by WPW syndrome in the context of MI and highlights the importance of using high-sensitivity troponin levels and echocardiography for early diagnosis to improve patient outcomes.

## Introduction

In 1930, a seminal article was published by Louis Wolff, Sir John Parkinson, and Paul Dudley White, describing 11 patients who experienced episodes of tachycardia associated with a sinus rhythm electrocardiographic (ECG) pattern of bundle branch block with a short PR interval. This condition was subsequently named Wolff-Parkinson-White (WPW) syndrome [1]. The prevalence of a WPW syndrome is estimated between 0.07-0.25 percent in the general population [2,3]. Diagnosing myocardial infarction (MI) via ECG in patients with WPW syndrome is often challenging because the abnormal activation sequence seen in WPW syndrome can obscure the typical ECG findings of MI [4-6]. The presence of Q waves can often be misleading in patients with pre-excitation and potential infarction. Additionally, the visible T-wave abnormalities in these patients may resemble the clinical ECG changes associated with acute coronary syndrome (ACS) [7,8,9]. In this report, we present the case of a male patient diagnosed with WPW syndrome who experienced percutaneous coronary intervention (PCI) due to non-ST-elevation myocardial infarction (NSTEMI). We showcase the clinical findings observed to emphasize the importance of early myocardial injury diagnosis in patients with WPW syndrome.

## Case presentation

A 39-year-old Caucasian male patient without a history of chronic disease or medication presented with chest pain to a family health center clinic. The condition began one week ago with intermittent mild chest pain localized to the left side, characterized by a burning and dull ache, with no increase or decrease in intensity and no associated symptoms. On the day the patient came to health center, the patient experienced increased pain accompanied by palpitations with mild sweating. Initial vitals showed blood pressure of 126/78 mmHg, heart rate of 83 beats per minute, respiratory rate of 20 breaths per minute, SpO2 of 100%, and an ear temperature of 36.8°C.

On general examination, the cardiovascular system revealed no raised jugular venous pressure, no murmurs, normal S1 and S2 heart sounds, and no pedal oedema. The respiratory system examination showed equal and bilateral normal breath sounds. The abdominal examination indicated a soft and non-tender abdomen. 

**Figure 1:**
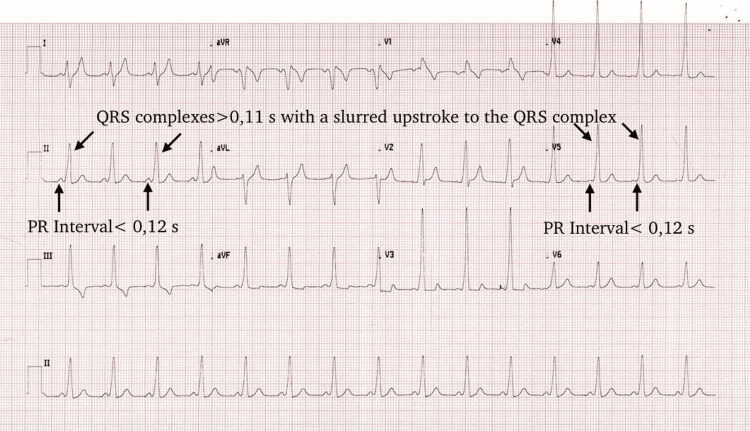
ECG of the patient at the Family Health Center PR interval<0,12 s, QRS complexes>0,11 s with a slurred upstroke to the QRS complex (ECG print specifications 25 mm/s, 10 mm/mV)

Initial ECG of the patient displayed findings comparable with WPW pattern with sinus rhythm Type A pattern (Figure 1). Short PR interval and prolonged QRS complexes with a slurred upstroke to the QRS complex were noted in ECG. 

**Figure 2:**
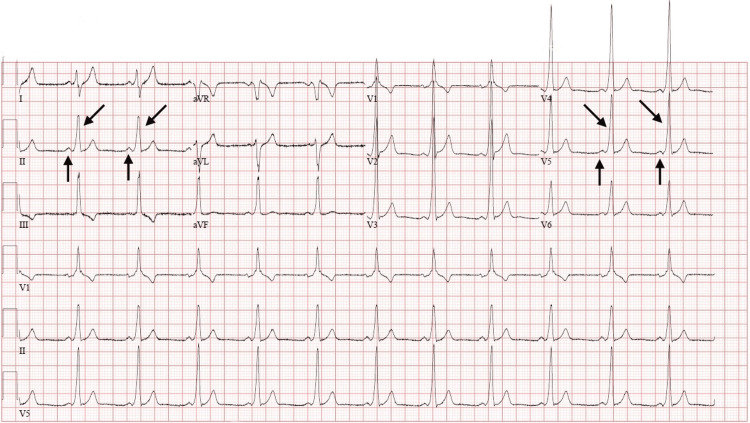
ECG of the patient at the Tertiary Hospital Similar to the ECG in Family Health Center, no ST segment or T wave changes, short PR segment, prolonged QRS with slurred upstroke to QRS complexes.

In the presence of typical chest pain and a WPW pattern on the ECG, the patient was referred to the tertiary hospital emergency department by ambulance. The patient was evaluated in the tertiary hospital emergency department, blood tests were ordered, and an ECG was performed again (Figure 2). There were no ECG changes.

When the blood tests were completed, the hemogram values were within the normal range (NR). Renal function tests and mineral levels were also within normal range: urea 20 mg/dl (NR: 7-43 mg/dl), creatinine 1.04 mg/dl (NR: 0.7-1.3 mg/dl), potassium 4.4 mg/dl (NR: 3.1-5.1 mg/dl), sodium 139 m/mol (NR: 136-146 m/mol), calcium 9 mg/dl, chlorine 100 mg/dl (NR: 98-107 mg/dl).

The troponin T level was elevated at 495 ng/ml (NR: 0-0.033 ng/ml). A repeat troponin test done 4 hours later showed a level of 485 ng/ml.

The patient was referred to a cardiology consultant, who decided to admit the patient to the cardiology critical care ward. An ECG was taken again, and there were still no changes. Echocardiography was performed on the patient (Table 1).

**Table 1:** Echocardiography Report LV: Left Ventricle; LVEF: Left Ventricular Ejection Fraction

Echocardiography Report	
Left Ventricle	Normal size left ventricle. Normal global systolic LV function.
	Biplane LVEF is calculated at 54%.
	Regional wall motion abnormalities.
	The basal inferoseptal, basal inferior, basal inferolateral and mid inferolateral left ventricular wall segments are hypokinetic.
	All remaining scored left ventricular wall segments are with no wall motion abnormalities.
	Grade 1 diastolic dysfunction (normal Left Atrium pressure).
	Normal left ventricular wall thickness.
	No obvious intracardiac thrombi are identified in the left ventricle.
Right Ventricle	Normal size right ventricle. Normal Right Ventricle function.
Left Atrium	The left atrium is normal in size.
Right Atrium	The right atrium is normal in size.
Mitral Valve	Normal Mitral Valve morphology.
Aortic Valve	Normal Aortic Valve morphology and function.
Tricuspid Valve	Mild tricuspid regurgitation. Pulmonary artery pressure is normal.
Pulmonic Valve	Normal morphology and function.
Aorta	No dilatation of the aorta.
Great Vessels, Vena Cava Inferior	Normal size and course of the Vena Cava Inferior
Pericardium	No pericardial effusion.

Afterwards, a coronary angiography was recommended and performed. There were ectasia in vessels with slow flow and no obstructed vessels (Table 2).

**Table 2:** Coronary Angiography Report

Coronary Angiography Report	
Left Main (LMS)	Normal
Left Anterior Descending (LAD)	Normal
Left Circumflex (LCX)	Normal
Right Coronary Artery (RCA)	Normal
Result	Ectasia in vessels with slow flow

The day after angiography done, a Holter ECG was performed (Table 3).

**Table 3:** Holter ECG report

Holter ECG Report				
General			Heart Rate Data	
QRS complexes	113368		Maximum HR(avg for 1 min)	120
Venticuler ectopic beats (0,00 %)	4		Average	85
Supraventricular ectopic beats (0,01%)	11		Minimum heart rate	55
Total time as classifed as noise	0%		Beats in Tachycardia (16,55%)	18766
Atrial fibrilation episodes	0		Beats in Bradycardia (1,74%)	1976
Minutes of atrial fibrilation	0		Seconds max R-R	1,17
Supraventricular Ectopy (0,01%)			Ventricular Ectopy(0,00%)	
Isolated	11		Isolated	4
Couplets	0		Couplets	0
Triplets	0		Triplets	0
Bigeminal cycles	0		Bigeminal Cycles	0
Runs(≥ 4 beats) totaling 0 beats, 0%	0		Runs(≥ 4 beats) totaling 0 beats, 0%	0
Tachycardia (≥ 3 beats at 120 bpm) totaling 0 beats, 0%	0		Tachycardia (≥ 4 beats at 120 bpm) totaling 0 beats, 0%	0
Bradyarrhythmia				
Pause (>2.00 s)	0			
N-N Delay (Delay > 140%)	0			

Upon completing all ward investigations, the patient was discharged with medication and instructions for cardiology outpatient follow-up. The final diagnosis is non-ST MI with WPW.

## Discussion

Early recognition of MI in WPW patients is extremely important. It may facilitate timely intervention, reduce the risk of complications and improve patient outcomes. Delayed diagnosis may lead to delayed treatment, increase the risk of myocardial damage and therefore increase mortality. 

Recognizing MI early may be difficult in WPW because WPW can either mimic the appearance of MI or obscure the electrocardiographic abnormalities indicative of an acute MI. Positive delta waves can cover up necrotic Q waves while ischemic ST-T wave changes and injury-induced modifications might be concealed by secondary repolarization changes because of the WPW pattern. Accurate diagnosis depends on clinical symptoms plus serial levels of myocardial biomarkers [9]. 

De Castro et al. reported a 57-year-old male patient where concealed WPW was revealed following an acute coronary syndrome. This case highlights the importance of considering WPW in differential diagnosis and highlights the potential for WPW in complicating MI diagnosis [10].

Verani et al. report a 49-year-old male case report with WPW syndrome presented with MI symptoms and the diagnosis was confirmed through ECG changes [4]. However, performing serial ECGs alone is time-consuming and can delay the diagnosis of MI, consequently postponing the primary coronary intervention necessary for myocardial salvage. Additionally, identifying ST elevation in patients with WPW syndrome is challenging due to the presence of secondary ST-T changes.

Chin et al. report a 60-year-old male patient with a WPW pattern on ECG who presented with NSTEMI. Despite normal initial cardiac troponin levels, follow-up tests revealed elevated troponin I levels. The patient had a successful percutaneous coronary intervention and radiofrequency catheter ablation for his accessory pathway. This case shows the importance of serial biomarkers in diagnosing MI in WPW [11].

## Conclusions

In our case, troponin levels were assessed through laboratory tests because of ongoing chest pain and the presence of a WPW pattern on the ECG. Early echocardiography was conducted in conjunction with a prompt cardiology consultation, revealing wall motion abnormalities. In conclusion, our report demonstrates that the WPW pattern can obscure the presence of myocardial infarction in patients presenting with chest pain. Early diagnosis using high-sensitivity troponin levels and echocardiography can significantly improve the chances of survival.
